# Chikungunya clinical management guidance: insights from a health system stakeholder study in Indonesia

**DOI:** 10.1136/bmjgh-2025-019419

**Published:** 2026-02-18

**Authors:** Evi Sukmaningrum, Komang Ayu Kartika Sari, Theresia Puspoarum Kusumoputri, Ni Luh Putu Ariastuti, Kevin Kristian, Eric Sindunata, Hendry Luis, Pande Putu Januraga, Peter W Horby, Louise Sigfrid, Keerti Gedela

**Affiliations:** 1 Faculty of Psychology, Atma Jaya Catholic University of Indonesia, South Jakarta, DKI Jakarta, Indonesia; 2 Center of Excellence for Health Policy and Social Innovation HIV AIDS Research Center, Atma Jaya Catholic University of Indonesia, South Jakarta, DKI Jakarta, Indonesia; 3 Centre for Public Health Innovation, Udayana University Faculty of Medicine, Denpasar, Bali, Indonesia; 4 School of Medicine & Health Sciences, Atma Jaya Catholic University of Indonesia, North Jakarta, Special Capital Region of Jakarta, Indonesia; 5 Yayasan Bali Peduli, Bali, Indonesia; 6 ISARIC Global Support Centre, Pandemic Sciences Institute, Oxford University, Oxford, UK; 7 ISARIC Global Support Centre, Pandemic Sciences Institute, Nuffield Department of Medicine, Oxford University, Oxford, Oxfordshire, UK; 8 Policy and Practice Research Group, University of Oxford, Oxford, UK; 9 Chelsea and Westminster Hospital NHS Foundation Trust, London, UK

**Keywords:** Health policy, Health systems evaluation, Public Health, Infections, diseases, disorders, injuries, Descriptive study

## Abstract

Chikungunya virus poses a significant public health challenge in Indonesia. Robust surveillance and diagnostics are lacking, and clinical management guidelines (CMGs) are absent.

This practice article draws on insights from a qualitative health system stakeholder study conducted in Jakarta and Bali, Indonesia. The study examined barriers to the development and implementation of chikungunya CMGs.

Findings reveal critical gaps in knowledge, diagnostic tools and prioritisation, exacerbated by systemic decentralisation and resource limitations. Robust, locally adapted CMGs could address these gaps, enhance clinical care and improve outbreak preparedness. This study underscores the importance of stakeholder engagement and capacity building in developing CMGs, offering lessons for low-resource settings managing emerging diseases.

**Summary box**

- Chikungunya is endemic in Indonesia but lacks clinical management guidelines (CMGs), resulting in inconsistent recognition and reliance on dengue protocols.
- Stakeholders revealed systemic barriers—low prioritisation, limited diagnostic capacity, decentralisation and insurance restrictions—yet expressed strong support for developing CMGs.
- Robust, locally adapted CMGs could standardise care, improve diagnostic accuracy and strengthen surveillance and outbreak preparedness.
- Indonesia’s experience highlights opportunities for other low-resource settings to address similar gaps in emerging infectious disease management.

## Introduction

 Chikungunya virus (CHIKV), a mosquito-borne arbovirus, has been endemic in Indonesia since the 1950s.^1^ Globally, chikungunya poses a significant health and economic burden, causing prolonged debilitating joint pain, reduced productivity and increased strain on healthcare systems.^2^ Recent outbreaks of chikungunya in 2025, including reported fatalities, highlight its continuing global public health threat.^3^

Despite regular outbreaks and significant health and socioeconomic impacts, chikungunya remains a neglected public health issue in Indonesia and across Asia.^4 5^ Current prevention and control guidelines in Indonesia, issued in 2012 and 2017,^6^ focus primarily on community-level interventions and lack clinical guidance for healthcare providers. Clinical management guidelines (CMGs) are important tools to guide clinical decision-making. They facilitate the standardisation of evidence-based healthcare, improve patient outcomes and prevent unnecessary management associated with risk or harm.^5^ Their absence in Indonesia results in inconsistent care, reported mismanagement, prolonged suffering and poor surveillance.

The re-emergence of CHIKV in urban and rural settings in Indonesia highlights the need for comprehensive public health responses.^5 6^ Developing CMGs tailored to Indonesia’s healthcare system would provide clear clinical pathways, improve patient management and outcomes and strengthen outbreak preparedness. Establishing these guidelines is critical to ensuring a more systematic and equitable approach to chikungunya care across diverse healthcare settings in the country.

A qualitative health system stakeholder study was conducted in the Jakarta region and Bali provinces to address these challenges. This study aimed to understand knowledge, attitudes and practices (KAP) related to chikungunya and identify barriers and facilitators to CMG development and implementation. This study was part of a wider multinational project to address the critical gaps in the availability, inclusivity, scope and implementation of CMGs for high-priority infectious diseases with epidemic and pandemic potential.^7^ This work contributes to efforts to inform evidence-based healthcare standardisation and frameworks for future guideline development and implementation during infectious disease outbreaks.^5 7^

### Study purpose and methodology

A qualitative, in-depth case study analysis was conducted from March to October 2022 in the Indonesian provinces of Jakarta and Bali. Jakarta is an urban and suburban setting, while Bali is a rural setting. The case studies were conducted to understand the implementation of CMGs for diagnosing and managing chikungunya in outpatient, community and hospital settings. They specifically explored stakeholders’ perceptions and experiences regarding chikungunya and its management within the Indonesian healthcare system.

The objectives were to:

1. Assess stakeholders’ knowledge of CHIKV, chikungunya disease and existing guidelines.
2. Identify gaps and challenges in diagnosing and managing chikungunya cases.
3. Understand stakeholder attitudes towards CMGs, barriers to their development and implementation and perceived impact on patient care.

#### Design and setting

**Figure 1:**
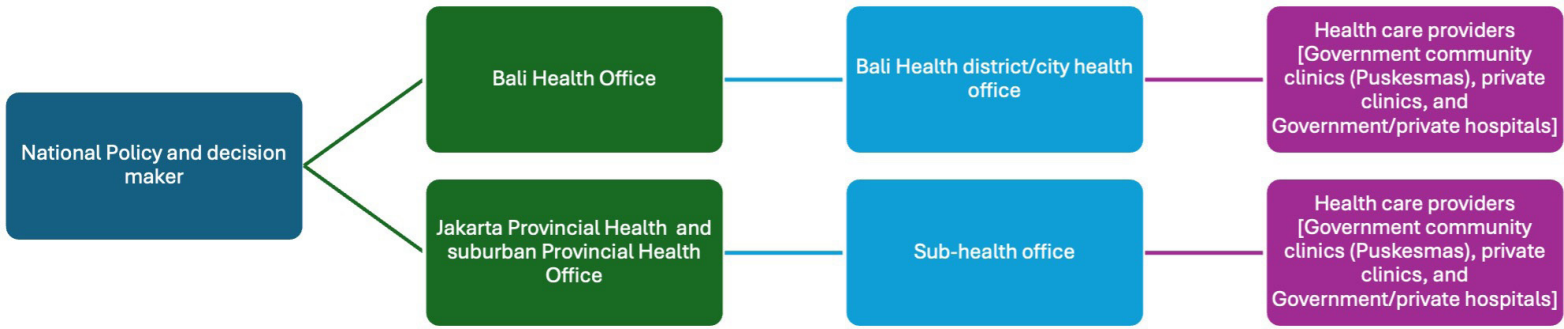
Stakeholder mapping for participants in Indonesia (attached as a JPEG file).

Jakarta and Bali provinces are diverse regions with distinct differences. They were selected for their differing healthcare systems and governance (eg, provincial vs district governance), population demographics and environmental diversity. Participants were recruited using purposive sampling techniques based on stakeholder mapping across the health system and recommendations from the health province in Jakarta and the health district in the Bali region (figure 1). Data collection was conducted through interviews and focus group discussions (FGD).

#### Study sample

This study involved 92 key informants from various stakeholders, representing a broad range of stakeholders in Indonesia’s healthcare system. Stakeholders included representatives and clinical staff from the Ministry of Health, provincial and district health offices, public and private healthcare providers and community health centres. Healthcare providers included doctors, nursing staff and programme manager administrators.

The breakdown of participants is as follows:

- *National stakeholders*: three representatives from the Ministry of Health (table 1).
- *Jakarta Province (urban and suburban sites)*: there were 43 participants, including senior and clinical staff from the health province and sub-health district (suku dinas) health offices, community health centres and hospital healthcare providers (table 2).
- Bali Province (rural site): there were 46 participants, including senior health official staff from the Bali Provincial, City and District Health Offices and community and hospital healthcare providers (table 3).

**Table 1:** Profile of national participants

Type of stakeholder	Interview
Ministry of Health	1
WHO	1
PAPDI	1

PAPDI, Perhimpunan Dokter Spesialis Penyakit Dalam Indonesia.

**Table 2:** Profile of Jakarta and suburban site participants

Subsites	Type of stakeholder	Data collection		Number of participants
		Interview	Focus group discussions	
Jakarta	Provincial Health Office (senior health official)	1		1
	Subprovincial Health Office (health officials)		1	5
	Healthcare providers (doctors, nursing staff and public health officials)			
	PHC—doctors		5	15
	Private hospital—doctors		1	3
	Public health and senior programme representatives at PHC		1	7
Bogor	District Health Office (senior health official)	1		1
	Healthcare providers from the community health centre		1	3
Depok	District Health Office (senior health official)	1		1
	Healthcare providers from community health centre		1	3
Bekasi	District Health Office (senior health official)	1		1
	Healthcare providers from the community health centre		1	3
	Total	4	10	43

PHC, public health centre.

**Table 3:** Profile of Bali site participants

Subsites	Type of stakeholder	Data collection		Number of participants
		Interview	Focus group discussions	
Denpasar	City Health Office (senior health officials)	3		3
	Healthcare providers (doctors, nurses and programme managers, administrative representatives)			
	Public hospital (doctors and nursing staff)	2	1	7
	Private hospital (doctors and nursing staff)	2	1	7
	Private clinic (doctors and nursing staff)		1	4
	Public health centre (public health and senior programme representatives)		1	4
Badung	District Health Office (health officials)	3		3
	Healthcare providers (doctors, nursing staff and programme manager administrators)			
	Public hospital (doctors and nursing staff)	2	1	7
	Private hospital (doctors)	2		2
	Private clinic (doctor)	1		1
	Public health centre (doctors, nursing staff and programme manager administrators)		1	4
Others	Bali Province Health Office (health officials)	3		3
	Clinical infectious diseases researcher from Udayana University, Bali	1		1
	Total	19	6	46

### Data collection

Semistructured interviews and FGDs were conducted with 92 participants.

### Cross-site coding and consolidation

Data collection involved purposive sampling and qualitative thematic framework analysis using NVivo 12 software. Interviews were transcribed, anonymised and coded to identify recurring themes related to KAP, barriers and facilitators for CMG development. Rigorous and inter-rater coding reviews were applied to validate findings.^8^

After initial coding within site teams (Jakarta and Bali), transcripts and emergent themes were reviewed by the full research group during cross-site meetings. Internal coding validity checks were conducted to address subjective bias and ensure consistency. Researchers from both teams participated in consensus discussions to reconcile differences and refine categories, thus achieving a consolidated thematic framework that captured both common and site-specific findings. This process ensured that variations by site were retained and highlighted within the broader analysis. It also enabled discussions for feedback on results to country- and local-level clinical and policymaking teams in Indonesia.

### Rationale for consolidated thematic analysis

Indonesia’s clinical guideline development for infectious diseases is governed nationally, with the Ministry of Health Regulation No. 1438/MENKES/PER/IX/2010 mandating the use of National Guidelines for Medical Practice (PNPK) and standard operating procedures (SOPs). This framework ensures that clinical management guidance is developed centrally and subsequently disseminated for implementation throughout diverse provincial and district health systems. For this reason, our analytic approach used an overarching, consolidated thematic analysis to synthesise data from both Jakarta and Bali, focusing on insights that inform national CMG policy and practice. While this approach respects site variations, it recognises that local adaptation currently operates within a regulated, top-down system, supporting the relevance of a unified analysis for national guideline translation.

These results have since been presented and discussed with Indonesian national senior stakeholders and UK policymaking and funding teams (representatives from the Wellcome Trust, Oxford University, the WHO and allied international teams from this group).

## Results

### Knowledge and practices

Stakeholders demonstrated varying levels of knowledge about chikungunya. While most were familiar with CHIKV, they had limited knowledge of the disease and its clinical management. Due to the absence of local CMGs, healthcare providers often relied on generic fever protocols, dengue guidelines or international resources (eg, WHO and Centres for Disease Control and Prevention (CDC)).

In Bali, recent suspected outbreaks heightened awareness, with some providers using informal networks (eg, WhatsApp groups) to share information and relying on patients providing suspicion of chikungunya rather than clinical acumen. Diagnostic challenges were widespread, with most stakeholders citing limited access to PCR or serology tests. Financial barriers further exacerbated these challenges, as diagnostic tests were often not covered by basic health insurance, which also prevented chikungunya from being entered as a possible diagnosis on health records.

I haven’t seen a clinical management guideline up to now. There is no updated guideline; it’s not yet available. FGD with District Health Offices of DKI Jakarta

Well, for our work, we lean more toward SOPs; it’s more like a technical guideline, Sir, it’s a procedure for our day-to-day work, so it’s actually more practical to refer to an SOP. FGD with Puskesmas in DKI Jakarta

There is none (referring to a CMG for chikungunya). For Chikungunya, I get my information from the courses that we took at school, at the university. FGD with Puskesmas in DKI Jakarta

After 20 years of practice, it was the first time I found chikungunya suspect and I was aware of the diagnosis because the patient told me so…they have families with similar symptoms and other doctors said it was chikungunya. Interview with the head of a private clinic in Badung, Bali

We use the approach for fever, so for acute febrile illness we usually refer to do a routine blood test to see whether there is lymphopenia or thrombocytopenia as the sign of a viral infection. So the standard is conducting a serial blood test and if there is another problem, then we will have other tests as well. Internist at a private hospital in Badung, Bali

### Attitudes towards CMGs

Stakeholders expressed positive attitudes towards developing chikungunya-specific CMGs. Key reasons included:

- Providing clear diagnostic and treatment pathways.
- Enhancing provider confidence in managing cases.
- Standardising care to improve patient outcomes.

However, the perceived low severity of chikungunya and its similarity to but lower clinical importance than dengue were cited as barriers to prioritising CMG development. Participants emphasised that increasing case numbers or outbreaks would likely drive the urgency of implementing the guidelines.

For me, because there are a number of suspected cases, it is important to have guidelines for diagnosis and treatment, also to prevent outbreak. I am afraid if we are not ready and then suddenly it becomes an outbreak. Interview with the head of puskesmas in Denpasar, Bali

Yes, it’ll certainly be better if they are differentiated (referring to guidelines for chikungunya as separate from dengue) since there has to be a separate procedure for DHF, like we said earlier it has different levels, there is the shock syndrome so there has to be a pathway for managing the shock. But chikungunya doesn’t have it, so if there is a PPK (referring to the technical SOP), a clinical practice guideline that is specific for chikungunya, it’ll be better. FGD with South Bogor Puskesmas

In my opinion, it is necessary because we can be more certain, this is dengue or chikungunya. I want to know what exactly distinguishes these two diseases, not just based on the symptoms; they are similar. Earlier, we could be like this patient. Explain the disease to the patient, oh no, this person is more specific here, so we can talk about certainty, better explanation to the patient like that. FGD with Public Health Centre, South Denpasar, Bali

So if there is a guideline and it’s turned into an SOP, yes that will definitely be very useful for service delivery. It’ll primarily be for patient management, services will be more standard, and organized and perhaps quicker in managing each patient who has this illness following the SOP. FGD with Nurses of Puskesmas in DKI Jakarta

### Barriers to CMG development

1. Systemic decentralisation: Indonesia’s decentralised health system creates bureaucratic bottlenecks, complicating guideline development and dissemination. Local district health system governance allows local teams to finance and implement programmes autonomously. However, provincial health systems, such as in Jakarta, are more restrictive, preventing a coordinated nationalised and universal approach.
2. Resource constraints: limited diagnostic capacity, lack of mandatory reporting and insufficient epidemiological data hinder effective chikungunya management.
3. Low prioritisation: chikungunya is perceived as less severe than other infectious diseases, such as dengue fever, resulting in limited resource allocation and research interest. The focus on perceived high-priority diseases is also resource-constrained, exacerbating challenges to prioritise diseases such as chikungunya.

… We need a document that is of a higher level than a guideline…but I think it’s not about the guideline, but it’s about the commitment of all the stakeholders at the national and sub-national level in implementing the guideline. The root cause of the problem is lack of commitment, and it becomes very difficult to allocate resources, human resources as well as financial resources, at the provincial and district level for chikungunya programme implementation. Interview with WHO Indonesia

So…PNPK (National CMG) should be developed by Directorate of Health Services. There are priorities and how high the priority of dengue that makes the PNPK has just been developed in 2021. Dengue has been there since 1968, but the PNPK has just been developed. We are from Directorate of Diseases Control, has outlined the need of PNPK for dengue several times, but due to the low priority, it was just developed last year. Interview with MoH

From the latest guidelines, we have a problem in determining the diagnosis of chikungunya. Doctor, because to be able to answer whether this is really diagnosed with chikungunya, we need a high-tech PCR lab from that serum, that’s what the service and puskesmas have not been able to do. Interview with Head of Diseases Prevention and Control, Badung District Health Office, Bali

### Facilitators for CMG implementation

1. Stakeholder engagement: the inclusive involvement of clinicians, public health officials and community representatives can ensure that guidelines address local needs.
2. Capacity building: training programmes to improve provider knowledge of chikungunya diagnosis, treatment and reporting can strengthen health systems.
3. Integrated surveillance: incorporating chikungunya into existing surveillance systems and subsidising diagnostics through insurance schemes can improve case detection and reporting.

A summary of consolidated findings is presented in table 4. For details on site-specific findings, please see online supplemental table 5.

**Table 4:** Summary of consolidated key findings

Theme	Subcategory	Findings
Knowledge	Awareness of chikungunya	Participants demonstrated basic awareness of chikungunya but detailed knowledge of transmission, diagnosis and treatment was limited. Most clinical stakeholders used general local protocols for fever or dengue, individual knowledge and experiences and other international guidelines from Centres for Disease Control and Prevention or WHO. Clinical stakeholders admitted that patients were often the first to suspect chikungunya, exposing the lack of knowledge. There was awareness that the lack of diagnostics meant disease burden was poorly understood. Stakeholders acknowledge that confirming outbreaks was challenging because clinicians are unable to establish diagnoses in suspected cases.
	Familiarity with current guidelines, lack of guidelines.	There are no dedicated national or local CMGs for chikungunya. Existing guidelines (2012 and 2017) focus on chikungunya virus prevention and control at the community level and lack clinical management details. Dissemination of these guidelines was inconsistent.
Attitudes	Perception of chikungunya	Chikungunya is perceived as a low-priority disease due to its relatively mild presentation compared with dengue. Stakeholders acknowledged the need for guidelines but noted a low sense of urgency for their development. Key perceptions described to prioritise awareness and need for CMGs were increasing case numbers or when managing high-risk or complex cases.
	Need for CMGs	Stakeholders highlighted the importance of CMGs for providing clear diagnostic and treatment pathways, especially during outbreaks or when managing high-risk or complex cases. CMGs to aid the management of vulnerable populations with complex needs, such as pregnant women and the elderly, were recognised.
	Barriers to prioritisation	Systemic decentralisation, resource constraints, a lack of diagnostic tools and clinical importance were identified as barriers to prioritising chikungunya and developing CMGs. Chikungunya was not deemed a research priority from a national and international perspective.
Practices	Diagnosis and treatment	Diagnosis relied on clinical suspicion due to limited access to affordable PCR or serology tests. Practices for managing chikungunya cases were based on individual knowledge, fever standard operating procedures or international guidelines.
	Use of guidelines	Bali stakeholders reported greater use of informal networks (eg, WhatsApp groups) during outbreaks, while Jakarta stakeholders relied on general fever protocols.
	Reporting and surveillance	Surveillance and reporting mechanisms for chikungunya are inconsistent and not mandatory, leading to underreporting and an underestimated disease burden.
	Financial and administrative barriers	Insurance policies require definitive International Classification of Diseases, 10th revision codes, which do not include ‘suspected diseases’. This limits financial support for diagnostics and treatment, placing additional pressure on healthcare providers and patients. At the national level, there is a lack of systematic surveillance and diagnostic support, no requirement for mandatory reporting of suspected cases and a perceived lack of awareness of case-reporting procedures.

CMGs, clinical management guidelines.

## Discussion

The absence of national and local CMGs for chikungunya in Indonesia reflects systemic challenges within its decentralised health system. The responsibility for guideline development and dissemination is divided among various national and local health organisations, each with layers of management hierarchy. This structure creates bureaucratic bottlenecks, delaying the formulation and dissemination of essential clinical guidance. The existing control guidelines, last issued in 2017, focus exclusively on community-level prevention and control, leaving healthcare providers without clear clinical and supportive care protocols.

The lack of CMGs has contributed to inconsistent clinical knowledge and practice. Healthcare providers often rely on alternative resources, including guidelines for dengue, fever management SOPs and international recommendations from the WHO or CDC. This ad hoc approach exacerbates diagnostic delays, particularly as access to CHIKV-specific PCR or serology tests is limited and financially inaccessible for most patients. Insurance policies further complicate the situation by requiring International Classification of Diseases, 10th revision codes, which do not accommodate ‘suspected diseases’, restricting care options for acute and long-term consequences and preventing reporting. These systemic gaps highlight the urgent need for robust, locally adapted CMGs to improve patient care and the health system response. Guidelines must include clear diagnostic criteria, treatment protocols and strategies for managing vulnerable populations. Strengthened coordination between national and local health authorities is critical for efficient guideline development and dissemination.

Our choice of an overarching analytic approach is grounded in the national framework, which stipulates that infectious disease clinical management guidelines are developed, approved and disseminated through central mechanisms (MoH Regulation No. 1438/MENKES/PER/IX/2010). While Jakarta and Bali exhibit distinct local governance and outbreak histories, their input feeds into a single system for guideline adoption and scale-up. Our cross-case consolidation supports recommendations that inform national CMGs. The embedded quotes and table 4 demonstrate that key nuances and local practices, such as Bali’s outbreak-driven informal networks and Jakarta’s SOP-oriented routine, were identified and considered within this synthesis. This approach ensures the findings both reflect policy realities and incorporate regionally salient insights to maximise their relevance and utility for Indonesia’s health system.

## Recommendations

1. Develop locally adapted CMGs: CMGs should include clear diagnostic criteria, treatment pathways and protocols for managing vulnerable populations, such as pregnant women and older adults. These guidelines must be tailored to Indonesia’s resource constraints and epidemiological context.

Stakeholder investment: Directorate General of Disease Control and Environmental Health, Ministry of Health. Their role is central to issuing regulations on CMG development, overseeing dissemination and approving the guidelines for implementation across all provinces.

Medical professional associations, including the Indonesian Medical Association, the Indonesian Internal Medicine Specialist Association and the Indonesian Paediatric Society. Critical as clinical experts, contributing to the content development of the CMGs.

The Health Development Policy Agency (Badan Kebijakan Pembangunan Kesehatan/BKPK) can play a key role in evaluating the acceptability and feasibility of CMG development and implementation.

1. Strengthen coordination in decentralised systems: Establishing streamlined processes for CMG development and dissemination can reduce bureaucratic delays. Coordination mechanisms between national and local health authorities are crucial to ensure consistency and efficiency.

Stakeholder investment: While the Ministry of Health would establish a coordination mechanism between national and provincial levels, the Provincial and District Health Offices are responsible for integrating the CMG into local health strategies and implementation within regional health systems.

1. Enhance diagnostic capacity**:** Expanding access to affordable diagnostic tools, such as CHIKV PCR or serology tests, is critical. Subsidising diagnostics through national health insurance schemes can mitigate financial barriers and improve disease detection.

Stakeholder investment Equipment procurement would be facilitated through the Health Laboratory Centres (Laboratorium Kesehatan Masyarakat) and the Technical Implementation Units. These units can help provincial and district health offices provide access to CHIKV diagnostics for the community.

1. Prioritise chikungunya in public health agendas**:** Advocacy campaigns targeting policymakers and healthcare providers should emphasise chikungunya’s socioeconomic and health impacts. Increasing provincial, district and national health system awareness can elevate its priority in research funding and policy development.

Stakeholder investment: The National Directorate of Communicable Diseases plays a vital role in elevating priority on the national agenda.

1. Build provider capacity**:** Training programmes focused on chikungunya diagnosis, management and case reporting can enhance provider confidence and improve patient outcomes. Leveraging digital platforms to disseminate training materials can also increase accessibility.

Stakeholder investment: The Medical Professional Associations, in collaboration with universities and medical schools, play a vital role in developing curriculum for continuing medical education and continuing professional development to strengthen providers’ capacity and ensure implementation of CMGs in different health settings.

1. Integrate surveillance and reporting; Incorporating chikungunya into existing surveillance systems (such as for dengue fever) and making case reporting mandatory can provide more accurate data on disease burden, supporting evidence-based decision-making.

Stakeholder investment; The Sub-Directorate of Surveillance and Outbreak Response (Direktorat Surveilans dan Karantina Kesehatan) is responsible for providing evidence and technical assistance on surveillance systems.

1. Strengthen evidence through locally led research; Invest in research to evaluate the effectiveness of supportive care treatments, assess CMG implementation and identify optimal dissemination strategies. Generating robust evidence will help to close knowledge gaps, improve disease burden estimates and inform policy decisions in the context of Indonesia’s resource constraints and competing health priorities.

Several stakeholders from local research centres, universities and teaching hospital networks can be engaged to generate context-specific research and data generation.

## Conclusion

The absence of clinical management guidelines for chikungunya in Indonesia reflects systemic challenges in low-resource settings. Addressing these gaps requires coordinated efforts to prioritise chikungunya, engage stakeholders and build diagnostic and clinical capacity. Locally adapted CMGs can bridge knowledge and practice gaps, improving patient care and strengthening Indonesia’s health system. Lessons from this study offer valuable insights for other regions facing similar challenges with emerging infectious diseases.

By implementing these recommendations, Indonesia can overcome systemic barriers to effective chikungunya management, ultimately improving health outcomes and strengthening its healthcare system’s resilience to emerging diseases.

As described, the results have been presented to national and local policymakers in Indonesia, as well as UK partners and funders, to enable effective translation.

## Supplementary material

10.1136/bmjgh-2025-019419 online supplemental file 1

## Data Availability

Data are available upon reasonable request.

## References

[1] Nsoesie EO, Kraemer MU, Golding N (2016). Global distribution and environmental suitability for chikungunya virus, 1952 to 2015. Euro Surveill.

[2] Powers AM (2018). Risks of Chikungunya virus re-introduction. Lancet Infect Dis.

[3] European Centre for Disease Prevention and Control (2025). Chikungunya virus disease worldwide overview, situation update – july 2025. https://www.ecdc.europa.eu/en/chikungunya-monthly%20ECDC.

[4] Morrison TE (2014). Reemergence of Chikungunya virus. J Infect Dis.

[5] Webb E, Michelen M, Rigby I (2022). An evaluation of global Chikungunya clinical management guidelines: A systematic review. *eClinicalMedicine*.

[6] Kementerian Kesehatan Republik Indonesia (2017). Guidelines for vector control in indonesia.

[7] (2021). Report on gaps in clinical guidelines for emerging diseases, London (2021): Wellcome Trust Report..

[8] Creswell JW, Poth CN (2018). Qualitative inquiry and research design choosing among five approaches.

