# Supplementary material for: Chikungunya clinical management guidance: insights from a health system stakeholder study in Indonesia
Source: BMJ Glob Health. 2026 Feb 18;11(2):e019419. doi: 10.1136/bmjgh-2025-019419 (PMC12918689; doi:10.1136/bmjgh-2025-019419)
Supplement: online supplemental file 1 [file bmjgh-11-2-s001.docx]

**Table 5: Summary table of general site-specific findings**

| Theme | Jakarta (urban, suburban) | Bali (predominantly rural) |
| --- | --- | --- |
| Awareness and Knowledge | Basic awareness; greater reliance on SOP for fever and dengue | Outbreak-driven heightened awareness; informal WhatsApp group info sharing |
| Use of Guidelines | SOPs and general fever protocols preferred. Guidelines would support formal reporting structures. | Use of informal networks (WhatsApp). Increased referral to national guidelines during outbreak incidents. |
| Diagnostic Challenges | Limited access to PCR/serology which further impedes clinical suspicion. Clinical suspicion guides management but lack of understanding on disease presentation. | Limited access to PCR/serology which further impedes clinical suspicion. Patient-reported suspicion initiating diagnosis. |
| Reporting and Surveillance | Reporting mainly for confirmed dengue. Lack of mandatory CHIKV reporting and a confirmed diagnosis is needed for reporting. | Surveillance increases during suspected outbreaks, but reporting remains inconsistent |
| Perception of Urgency/Use of CMGs | Low urgency unless outbreak occurs. Guideline seen as nonessential due to perceived low caseload. | Increased sense of urgency during outbreaks. Stakeholders agree the need for a dedicated CMG. |
